# Impact of diabetes and glycemic status on ventricular–arterial coupling in the general population

**DOI:** 10.1186/s12933-025-02731-7

**Published:** 2025-04-18

**Authors:** Hannes Holm, Haris Zilic, Amra Jujic, Linda Johnsson, Gunnar Engström, Peter M. Nilsson, Carl Johan Östgren, David Kylhammar, Jan Engvall, Martin Magnusson

**Affiliations:** 1 Department of Clinical Sciences, Lund University, Malmö, Sweden; 2 Department of Cardiology, Skåne University Hospital, Malmö, Sweden; 3 Department of Clinical Physiology in Linköping, Linköping University, Linköping, Sweden; 4 Department of Health, Medicine and Caring Sciences, Linköping University, Linköping, Sweden; 5 Wallenberg Center for Molecular Medicine, Lund University, Lund, Sweden; 6 Hypertension in Africa Research Team (HART), North-West University Potchefstroom, Potchefstroom, South Africa

**Keywords:** Ventricular–arterial coupling, Diabetes mellitus, Pulse wave velocity, Global longitudinal strain, Cardiovascular risk

## Abstract

**Background/aims:**

Ventricular–arterial coupling (VAC) plays a crucial role in cardiovascular physiology, affecting cardiac function and arterial properties for optimal organ perfusion. Considering that diabetes mellitus (DM) is a known risk factor for incident heart disease and vascular damage, this study aims to investigate whether changes in VAC due to DM occur earlier, even before the onset of clinically evident cardiovascular disease in the general population.

**Methods:**

This retrospective study included 2,884 participants (mean age 57 years, 48% male) of the Swedish CArdioPulmonary BioImage Study (SCAPIS), where data on echocardiography and Pulse wave velocity (PWV) were available. Of these, 162 individuals (6%) had prevalent type 2 diabetes (DM), and 334 (12%) had prediabetes. VAC was quantified as the ratio of PWV to Global longitudinal strain (GLS). Linear regression models were used to assess associations between glycemic status (DM, prediabetes), HbA1c, fasting plasma glucose (fP-glucose), and VAC, adjusting for relevant covariates.

**Results:**

I the fully adjusted model, prevalent DM and the combination of DM and prediabetes were significantly associated with increased values of PWV/GLS (Beta = 0.28, *p* < 0.001 and Beta = 0.14, *p* < 0.001 respectively), while no significant association was found between prediabetes and PWV/GLS. Increasing values of HbA1c and fP-glucose were significantly associated with higher values of PWV/GLS (Beta = 0.01,*p* < 0.001 and Beta = 0.07,*p* < 0.001, respectively) signaling worse VAC. In participants without prevalent DM, higher HbA1c levels were linked to increased PWV/GLS in the age- and sex-adjusted model; however, this association was attenuated after further adjustment for additional confounders. Conversely, fP-glucose remained significantly associated with elevated PWV/GLS across all adjusted models.

**Conclusions:**

This study demonstrates a significant association between DM and impaired VAC, as reflected by elevated PWV/GLS, while no such link was observed in prediabetes. The transition from prediabetes to DM appears critical for VAC deterioration. Additionally, higher HbA1c and fP-glucose levels, even in non-diabetic individuals, were associated with worsened VAC, highlighting the impact of glycemic control on vascular function.

**Graphical abstract:**

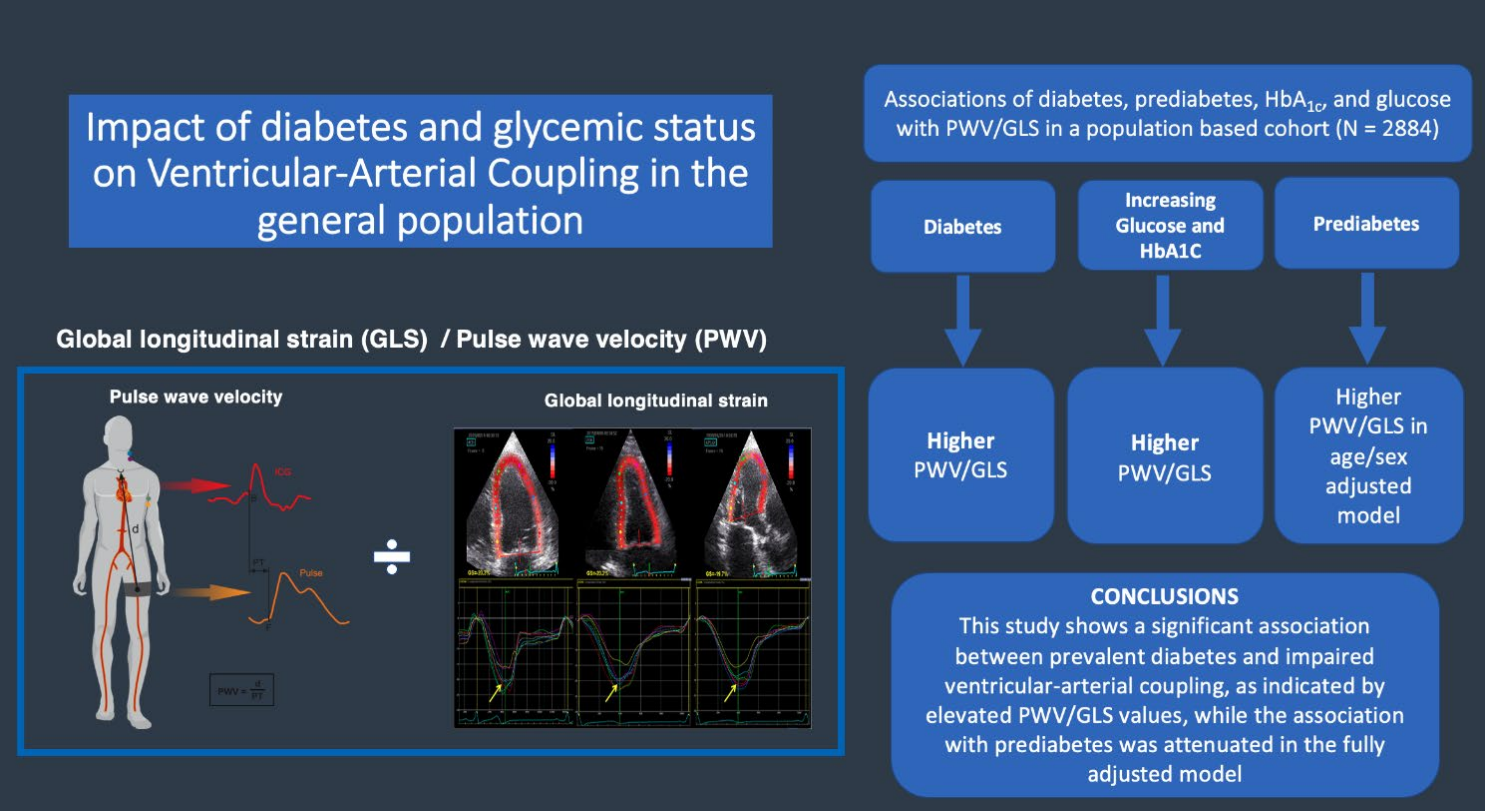

**Supplementary Information:**

The online version contains supplementary material available at 10.1186/s12933-025-02731-7.

## Introduction

Ventricular–arterial coupling (VAC) is fundamental to cardiovascular physiology, and reflects an intricate interplay between the heart and the arterial system that maintains optimal organ perfusion [1]. Impaired VAC is associated with increased cardiovascular risk. Traditionally, VAC has been quantified by the ratio of arterial elastance (E_a_) to ventricular elastance (E_es_), denoted as the E_a_/E_es_ ratio where E_a_ reflects the arterial load imposed on the left ventricle, while E_es_ represents the contractile properties and systolic stiffness of the left ventricle [2]. However, the optimal marker for assessing VAC in clinical practice remains a subject of debate, with various parameters proposed, including Pulse Wave Velocity (PWV)/Global Longitudinal Strain (GLS), which may offer advantages over traditional methods [3]. Although PWV/GLS may not fully capture the dynamic physiological interplay between ventricular contractility and arterial load as precisely as E_a_/E_es_, it provides a non-invasive, scalable alternative that reflects both arterial stiffness (via PWV) and myocardial systolic performance (via GLS), making it particularly suitable for large population studies. In direct comparison between E_a_/E_es_ and PWV/GLS, as observed in our previous work increasing PWV/GLS values are more closely associated with the extent of cardiovascular risk factors, emphasizing its potential as a valuable indicator in evaluating cardiovascular health [3]. Hence, the relevance of VAC extends beyond its role in normal cardiovascular physiology to its association with cardiovascular risk and the pathogenesis of cardiovascular diseases [4, 5] including heart failure [5], arterial hypertension [6], and degenerative aortic stenosis [7]. Despite a growing recognition of the importance of VAC in cardiovascular health and disease, limited data are available regarding whether disturbance in VAC is present in patients with diabetes mellitus (DM) or prediabetes. DM may impact VAC through both arterial and myocardial disease including increased arterial stiffness and endothelial dysfunction [8], resulting in increased arterial load on the left ventricle, and myocardial fibrosis and impaired contractility [9, 10]. There is also evidence that interventions that improve metabolic control and reduce cardiovascular risk factors in individuals with diabetes or prediabetes may potentially mitigate alterations in VAC and reduce the risk of cardiovascular complications [11]. We aimed to study whether VAC was associated with diabetes or prediabetes in a well-characterized population-based cohort, and to identify potential mechanisms underlying these associations.

## Methods

### Study population

**Fig. 1:**
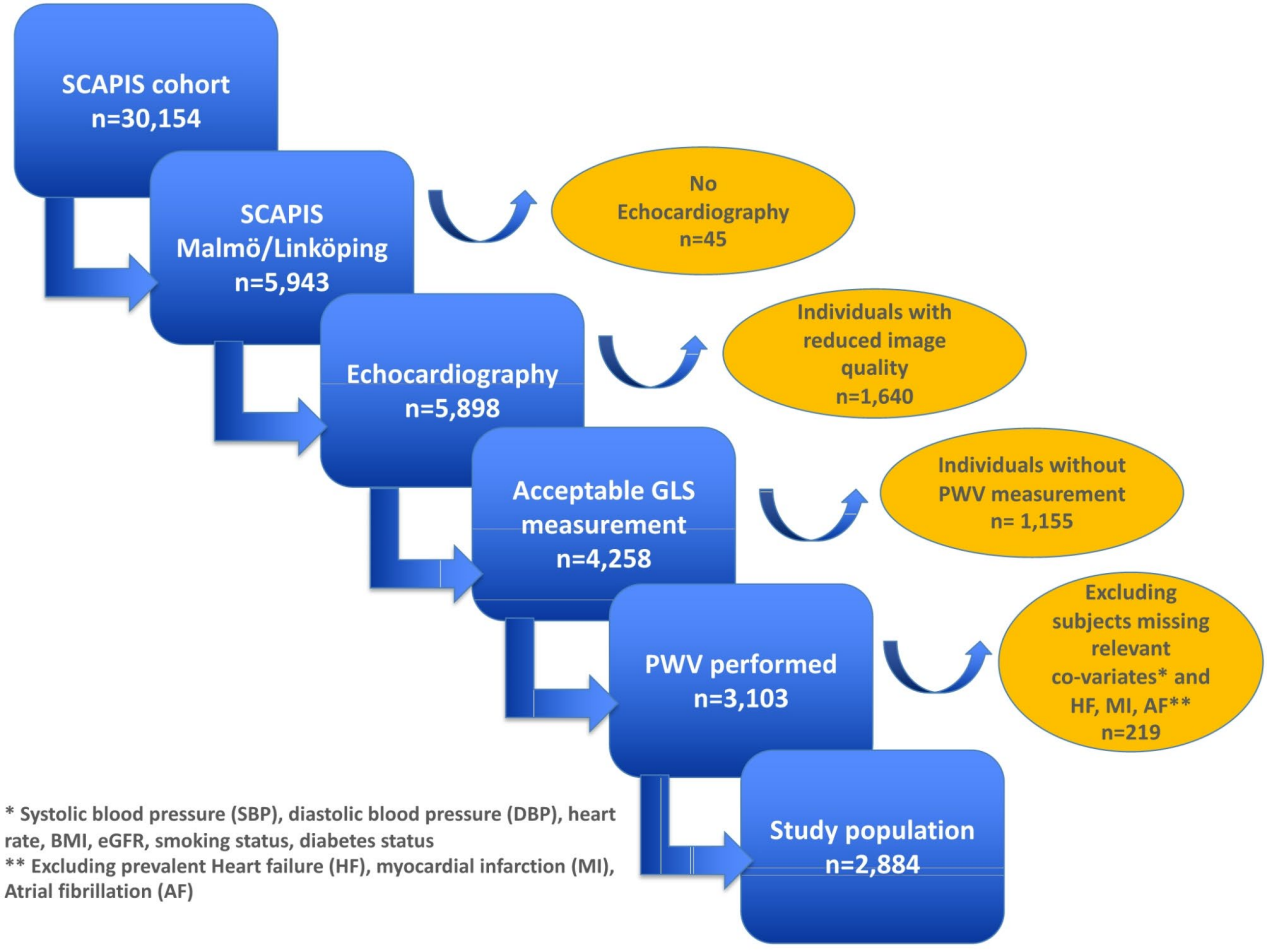
Study flowchart

Between 2013 and 2018, the Swedish CArdioPulmonary bioImage Study (SCAPIS) enrolled 30,154 individuals (age-range of 50–65 years, 51% women) from six cities in Sweden (Gothenburg, Linköping, Malmö/Lund, Stockholm, Umeå and Uppsala). The screening program was comprehensive and has been described elsewhere [12]. We included a subgroup of participants from the Malmö and Linköping sites who had contributed an echocardiography (*n* = 4,258) with GLS measurement. Of these, 3,103 subjects also had PWV measurements. Participants with reduced image quality, prevalent atrial fibrillation or flutter, heart failure, and myocardial infarction, or missing values on relevant covariates were excluded, resulting in a final study population of 2,884 individuals, Fig. 1. The study was approved by the Ethics Committee of Umeå University (number 2010-228-31 M) on behalf of all study sites and written informed consent was given by all participants. The study adheres to the Declaration of Helsinki.

### Clinical parameters

Blood samples were collected following an overnight fast. Standardized protocols at each site were used to measure fP-glucose, HbA1c, LDL and HDL cholesterol. Alere NTproBNP was analysed from frozen samples of fasting EDTA-plasma, using an Abbott Alinity I analyzer (Abbott Laboratories, Abbott Park, IL, USA). eGFR was calculated from determinations of serum creatinine using the modified CKD-EPI formula [13]. Blood pressure (mmHg) was measured twice in both arms after 5 min of rest in the supine position, using an Omron M10-IT automated oscillometric device (Omron Healthcare, Kyoto, Japan). The mean value of the two measurements for the arm with the highest mean systolic blood pressure was used. Hypertension was defined as a systolic blood pressure (SBP) ≥ 140 mmHg and/or diastolic blood pressure (DBP) ≥ 90 mmHg, and/or use of antihypertensive medication. Height (m) and body weight (kg) were measured by in light indoor clothing, and body mass index (BMI) was calculated as weight/height^2^. Information on life-style factors, medication and history of diseases was self-reported by a questionnaire. Smoking status was categorized as never, former, or current smoking. Carotid-femoral pulse wave velocity (c-f PWV), was assessed using the SphygmoCor Xcel system (AtCor Medical, Australia) [14]. The measurement protocol involved applanation tonometry to capture the arterial pressure waveform at the carotid artery, while a blood pressure cuff positioned on the thigh simultaneously recorded the femoral artery signal. Each assessment included two consecutive c-f PWV measurements; if the discrepancy between the two exceeded 0.5 m/s, a third measurement was performed to ensure accuracy and reproducibility.

### Classification of glycaemic status

Glycaemic status was classified based on fasting glucose levels, HbA1c, and/or self-reported diabetes. Participants were categorized as having normoglycaemia (fasting plasma glucose [fP-glucose] < 6.1 mmol/L and HbA1c < 42 mmol/mol), prediabetes (impaired fasting glucose with fP-glucose 6.1–6.9 mmol/L and/or HbA1c levels between 42 and 47 mmol/mol), or diabetes (fP-glucose ≥ 7.0 mmol/L, HbA1c ≥ 48 mmol/mol, and/or self-reported diabetes), following previously established criteria [15].

### Echocardiography

Transthoracic echocardiography (TTE) was performed by experienced sonographers using the GE Vivid E95 system equipped with M5Sc probes (GE Healthcare, Chicago, IL, USA) to assess cardiac morphology, left ventricular systolic and diastolic function, as well as valvular pathology. All measurements were conducted offline using EchoPAC software version 201 (GE Healthcare). Chamber size and function were measured in accordance with current guidelines [16, 17]. Mitral E- and A-wave diastolic inflow velocities were measured using pulsed wave Doppler ultrasound at the tips of the mitral leaflets. Early diastolic velocity (e’) at the basal septum was assessed using tissue Doppler imaging from the apical 4-chamber view with the Q-analysis tool in EchoPAC. The E/A ratio and E/e’ ratio were then calculated. Diastolic dysfunction was defined as a septal e’ < 7 cm/s and an E/e’ ratio > 14. Left ventricular hypertrophy (LVH) was defined as an indexed left ventricular mass > 95 g/m² for women or > 115 g/m² for men, while septal hypertrophy was defined as a septal wall thickness ≥ 10 mm in women and ≥ 11 mm in men [17]. Left ventricular ejection fraction (LVEF) was calculated using the modified Simpson’s method, while GLS was derived from the three apical views utilizing the semi-automated Automated Functional Imaging (AFI) module integrated within EchoPAC. Echocardiographic images underwent rigorous review, with cases excluded where endocardial visualization was inadequate in multiple segments, ensuring the reliability of strain data. VAC was defined as the ratio between PWV and GLS.

### Statistics

Continuous variables are presented as means (± standard deviation, SD), medians (25th-75th percentiles) or numbers (%). Differences in covariates are reported across quartiles of PWV/GLS and tested using one-way ANOVA test for normally distributed continuous variables, Mann-Whitney U-test for continuous variables with non-normal distribution, and χ2 test for binary variables. The PWV/GLS ratio was standardized and reported as standard deviations. We used linear regression models to study the association between DM/prediabetes and PWV/GLS using a three-step adjustment process as follows:1) unadjusted; 2) adjusted for age and sex, and 3) adjusted for age, sex, SBP, BMI, site, NTproBNP and LVEF. The same approach was used to assess the combined impact of DM and prediabetes on PWV/GLS. We also tested the association between HbA1c, fP-glucose and PWV/GLS in linear regression models with the same adjustment models. To assess whether glycemic control, including in the non-diabetic range, has an impact on VAC, we also analysed the association between HbA1c, fP-glucose levels and PWV/GLS in a subgroup including only individuals with normoglycemia. Post hoc analyses were performed using Tukey’s Honestly Significant Difference (HSD) test for continuous variables and Bonferroni-adjusted Z-tests for categorical variables to assess pairwise differences between subgroups of glycemic status and quartiles of VAC. There was no evidence of multicollinearity between the independent variables, as assessed using the variance inflation factor. All analyses were carried out using SPSS 28.0 (IBM, Chicago, IL, USA). A two-sided p-value below 0.05 was considered statistically significant.

## Results

### Population characteristics

In the full study sample (n = 2,884) 162 individuals had diabetes (6%), and 334 (12%) were prediabetic. The subjects with diabetes and prediabetes were more frequently male (p <.001) and had higher heart rates and BMI (both p <.001). There was also higher prevalence of hypertension and use of antihypertensive medications in subjects with diabetes (70% and 11%, respectively) compared to subjects with normoglycemia (35% and 3%) and prediabetes (47% and 5%) (p <.001). The LVEF was slightly lower in subjects with diabetes (58 ± 6%) compared to normoglycemia (60 ± 5%) (p =.002). In post hoc analyses, the PWV/GLS ratio is significantly higher in participants with DM compared to those with prediabetes and normoglycemia (both p < 0.001), with prediabetes also showing higher values than normoglycemia (p < 0.001). Compared to prediabetes and normoglycemia, participants with diabetes exhibited both significantly higher PWV and lower GLS values (both p < 0.001). indicating that the elevated PWV/GLS ratio in diabetes is driven by combined vascular and myocardial dysfunction, see Table 1. With increasing quartiles of PWV/GLS, a higher age (p < 0.001), higher height (p < 0.001), a higher proportion of men (p < 0.001), and a higher prevalence of hypertension (p < 0.001) were observed. Furthermore, with increasing quartiles of PWV/GLS, a significant upward trend was observed in BMI (p < 0.001), systolic blood pressure (p < 0.001), diastolic blood pressure (p < 0.001), heart rate (p < 0.001), HbA1c (p = 0.013), and fasting plasma glucose (p < 0.001). Conversely, eGFR (p = 0.002) and NT-proBNP (p < 0.001) demonstrated significant decreases with increasing quartiles of PWV/GLS. Left ventricular mass (p < 0.001) and E/e’ (*p* < 0.001) increased across quartiles of PWV/GLS whereas LVEF (*p* < 0.001) and septal E-velocity (*p* < 0.001) declined. The prevalence of DM and prediabetes increased from 4% to 9% in Q1 to 11% and 16% in Q4, respectively (both *p* < 0.001). The combined rate of prediabetes and DM increased from 13% in Q1 to 25% in Q4 (*p* < 0.001), Table 2.

**Table 1:** Population characteristics across glucometabolic status with post-hoc pairwise comparisons

Variables	Normoglycemia n = 2, 388	Prediabetes *n* = 334	Diabetes *n* = 162	*P*-value	Normoglycemia vs. Prediabetes (*p*)	Normoglycemia vs. Diabetes (*p*)	Prediabetes vs. Diabetes (*p*)
Male sex, n (%)	1109 (46)	167 (50)	106 (65)	< 0.001	0.666	< 0.001	0.003
Age, years (SD)	57 ± 4	58 ± 4	59 ± 5	0.600	0.010	< 0.001	0.053
Systolic blood pressure, mmHg (SD)	129 ± 17	133 ± 17	137 ± 16	0.315	< 0.001	< 0.001	0.094
Diastolic blood pressure, mmHg (SD)	81 ± 10	83 ± 10	83 ± 11	0.928	0.004	0.053	1.000
Heart rate, beats/min (SD)	60 ± 9	63 ± 10	66 ± 10	< 0.001	< 0.001	< 0.001	< 0.001
Body mass index, kg/m^2 (SD)	26 ± 4	28 ± 4	29 ± 4	< 0.001	< 0.001	< 0.001	< 0.001
Current smoker, n (%)	216 (9)	55 (17)	20 (12)	< 0.001	< 0.001	0.483	0.690
Hypertension, n (%)	827 (35)	158 (47)	114 (70)	< 0.001	< 0.001	< 0.001	< 0.001
Antihypertensive treatment, n (%)	79 (3)	15 (5)	18 (11)	< 0.001	0.801	< 0.001	0.018
Fasting plasma glucose, mmol/L (SD)	5.4 ± 0.4	6.1 ± 0.5	8.6 ± 2.9	< 0.001	< 0.001	< 0.001	< 0.001
HbA1c, mmol/mol (SD)	34 ± 3	38 ± 4	51 ± 15	< 0.001	< 0.001	< 0.001	< 0.001
NT-proBNP, ng/L (median [IQR], mean ± SD	48 [50], 62 ± 54	49 [47], 65 ± 75	41 [59], 59 ± 51	0.187	0.638	0.721	0.451
eGFR, mL/min/1.73 m^2 (SD)	75 ± 9	76 ± 10	79 ± 11	0.041	0.255	< 0.001	0.010
LDL-C, mmol/L	3.4 ± 0.9	3.4 ± 0.9	2.7 ± 1.0	< 0.001	0.319	< 0.001	< 0.001
HDL-C, mmol/L	1.7 ± 0.5	1.6 ± 0.5	1.5 ± 0.5	< 0.001	0.006	< 0.001	0.006
Ejection Fraction, % (SD)	60 ± 5	60 ± 5	58 ± 6	0.002	0.027	0.001	0.027
Left ventricular mass indexed (SD)	75 ± 19	76 ± 20	79 ± 20	0.337	0.743	0.029	0.198
PWV (SD)	10.4 (1.8)	10.7 (1.9)	11.3 (2.3)	< 0.001	0.040	< 0.001	< 0.001
GLS (SD)	19.5 (2.6)	19.1 (2.8)	18.4 (3.1)	< 0.001	0.013	< 0.001	0.027
PWV/GLS, (median [IQR], mean ± SD	0.53 [0.15], 0.54 ± 0.12	0.55 [0.16], 0.57 ± 0.12	0.61 [0.21], 0.63 ± 0.16	< 0.001	< 0.001	< 0.001	< 0.001

Values are presented as mean ± standard deviation for continuous variables and as number (percentage) for categorical variables

*eGFR* estimated glomerular filtration rate; *HbA1c* glycated hemoglobin; *NT-proBNP* N-terminal pro-B-type natriuretic peptide; *LDL* low density lipoprotein; *HDL* high density lipoprotein; *PWV* pulse wave velocity; *GLS* global longitudinal strain

**Table 2:** Population characteristics across quartiles of VAC (PVW/GLS)

	All *n* = 2884	Q1 *n* = 729 0.28–0.47	Q2 *n* = 726 0.47–0.53	Q3 *n* = 720 0.53–0.62	Q4 *n* = 709 0.62–1.25	*P*-value
Male sex, n (%)	1382 (48)	170 (23)	300 (41)	403 (56)	509 (72)	**< 0.001**
Age, years (SD)	57 (4)	56 (4)	56 (4)	57 (4)	58 (4)	**< 0.001**
Systolic Blood Pressure, mmHg (SD)	81 (10)	119 (14)	126 (14)	132 (14)	141 (17)	**< 0.001**
Diastolic Blood Pressure, mmHg (SD)	130 (17)	75 (8)	79 (9)	82 (8)	86 (10)	**< 0.001**
Heart rate, beats/min (SD)	60 (9)	58 (8)	59 (7)	60 (9)	62 (9)	**< 0.001**
Body Mass Index, kg/m^2 (SD)	26 (4)	25 (3)	25 (3)	26 (3)	27 (3)	**< 0.001**
Height, cm (SD)	172 (9)	168 (8)	172 (9)	174 (10)	176 (9)	**< 0.001**
Current Smoker, n (%)	291 (10)	82 (11)	70 (10)	70 (10)	69 (10)	0.695
Hypertension, n (%)	1099 (38)	137 (19)	207 (29)	286 (40)	469 (66)	**< 0.001**
Antihypertensive treatment, n (%)	112 (4)	32 (4)	25 (3)	21 (3)	34 (5)	**0.235**
*Classification of glycaemic status according to baseline examination results*						
Diabetes status 3 -levels						
Normoglycemia, n (%)	2388 (83)	638 (88)	622 (86)	599 (83)	529 (75)	**< 0.001**
Diabetes, n (%)	162 (6)	26 (4)	21 (3)	39 (5)	76 (11)	
Prediabetes, n (%)	334 (12)	65 (9)	83 (12)	82 (12)	104 (16)	
Diabetes status 2 -levels						
Normoglycemia, n (%)	2388 (83)	638 (88)	622 (86)	599 (83)	529 (75)	**< 0.001**
Diabetes or prediabetes, n (%)	496 (17)	91 (13)	104 (14)	121 (17)	180 (25)	
Fasting plasma glucose, mmol/L (SD)	5. 7 (1.1)	5.5 (0.7)	5.6(0.9)	5.7 (1.1)	6.0 (1.5)	**< 0.001**
HbA1c, mmol/mol (SD)	37 (8)	35(4)	35(5)	36 (6)	37 (8)	**< 0.001**
NT-proBNP, ng/L (SD)	60 (66)	70 (58)	60 (45)	59 (51)	59 (66)	**< 0.001**
eGFR, mL/min/1.73 m^2 (SD)	76 (9)	76 (9)	75 (9)	75 (8)	74 (9)	**0.002**
LDL-C, mmol/L	3.4 (0.9)	3.3 (0.9)	3.4 (0.9)	3.4 (0.9)	3.5 (1.0)	**< 0.001**
HDL-C, mmol/L	1.7 (0.5)	1.9 (0.5)	1.8 (0.5)	1.7 (0.5)	1.5 (0.5)	**< 0.001**
Ejection Fraction, % (SD)	60 (5)	61 (4)	60 (4)	59 (4)	57 (4)	**< 0.001**
Mitral valve E-wave velocity, cm/s	66 (15)	72 (15)	67 (15)	64 (14)	61 (14)	**< 0.001**
Septal e’, cm/s	6.9 (1.7)	7.7 (1.7)	7.2 (1.6)	6.7 (1.6)	6.1 (1.5)	**< 0.001**
E/e’, unitless	9.9 (2.9)	9.4 (2.5)	9.6 (2.8)	9.8 (3.0)	10.2 (3.0)	**< 0.001**
Left ventricular mass indexed (SD)	81 (20)	72(17)	73 (18)	77 (19)	81(20)	**< 0.001**
Left ventricular hypertrophy, n (%)	420 (15)	64 (9)	83 (11)	111 (15)	162 (23)	**< 0.001**
Septal Hypertrophy, n (%)	428 (15)	87 (12)	92 (13)	104 (15)	145 (21)	**< 0.001**
Septal e’ < 7, n (%)	181 (6)	36 (5)	38 (5)	45 (6)	62 (9)	**0.010**
E/e’ > 14, n (%)	200 (7)	32 (5)	45 (6)	59 (9)	64 (10)	**0.003**

*eGFR* estimated glomerular filtration rate; *HbA1c* glycated hemoglobin; *NT-proBNP* N-terminal pro-B-type natriuretic peptide; *e’* Early diastolic mitral annular velocity; *E* Early mitral inflow velocity; *LDL* low density lipoprotein; *HDL* high density lipoprotein. Bold indicates statistical significance at *p* < 0.05

### Association between VAC, HbA1c and diabetes/prediabetes

Diabetes was independently associated with increased PWV/GLS, after full adjustment (Beta = 0.28, 95% CI: 0.17–0.39, *p* < 0.001). With the full adjustment, there was a non-significant association between prediabetes and PWV/GLS in the same direction (Beta = 0.07, 95% CI: -0.01 to 0.15, *p* = 0.075). Higher levels of both HbA1c and fP-glucose were also significantly associated with increased PWV/GLS (Beta = 0.01, 95% CI: 0.01–0.02, *p* < 0.001 and Beta = 0.07, 95% CI: 0.04–0.09, *p* < 0.001, respectively). In a fully adjusted linear regression model using ordinal coding of glycemic status (0 = normoglycemia, 1 = prediabetes, 2 = diabetes), a significant positive trend was observed in relation to VAC (B = 0.07, *p* < 0.001), Table 3. In participants without diabetes, HbA1c was significantly associated with PWV/GLS in no adjustment model (Beta = 0.01, 95% CI: 0.002–0.02, *p* = 0.016) and after age/sex adjustment (Beta = 0.01, 95% CI: 0.001–0.02, *p* = 0.029). However, after fully adjustments, the association was no longer significant (Beta = 0.005, 95% CI: -0.003–0.013, *p* = 0.218). In contrast, fP-glucose remained significantly associated with higher PWV/GLS across all adjustment models, Table 4.

**Table 3:** Linear regression results for the associations of diabetes, prediabetes, HbA1c, and glucose with PWV/GLS

Variables	Unadjusted		Age/sex adjusted		Fully adjusted	
	Beta, 95% CI	*p*-value	Beta, 95% CI	*p*-value	Beta, 95% CI	*p*-value
Diabetes	0.63 (0.49–0.77)	**< 0.001**	0.45 (0.32–0.58)	**< 0.001**	0.28 (0.17–0.39)	**< 0.001**
Prediabetes	0.20 (0.10–0.30)	**< 0.001**	0.15 (0.06–0.25)	**0.001**	0.07 (-0.01 to 0.15)	0.075
Prediabetes + diabetes	0.35 (0.26–0.43)	**< 0.001**	0.26 (0.18–0.34)	**< 0.001**	0.14 (0.07–0.21)	**< 0.001**
HbA1c	0.03 (0.02–0.03)	**< 0.001**	0.02 (0.01–0.02)	**< 0.001**	0.01 (0.01–0.02)	**< 0.001**
fP-glucose	0.18 (0.12–0.21)	**< 0.001**	0.12 (0.09–0.15)	**< 0.001**	0.07 (0.04–0.09)	**< 0.001**
Glycemic status	0.17 (0.031–0.047)	**< 0.001**	0.13 (0.02–0.04)	**< 0.001**	0.07 (0.01–0.02)	**< 0.001**

*Fully adjusted model*: Adjusted for age, sex, systolic blood pressure, BMI, study site, NT-proBNP, and left ventricular ejection fraction (LVEF). Bold indicates statistical significance at *p* < 0.05

**Table 4:** Linear regression results for the associations of HbA1c and fP-glucose with PWV/GLS in individuals without diabetes

Variables	Unadjusted		Age/sex adjusted		Fully adjusted	
	Beta, 95% CI	*p*-value	Beta, 95% CI	*p*-value	Beta, 95% CI	*p*-value
HbA1c	0.01 (0.002–0.022)	**0.016**	0.01 (0.001–0.020)	**0.029**	0.01 (-0.003-0.013)	0.218
fP-glucose	0.17 (0.107–0.227)	**< 0.001**	0.11 (0.058–0.170)	**< 0.001**	0.05 (0.003–0.097)	**0.038**

*Fully adjusted model*: Adjusted for age, sex, systolic blood pressure, BMI, study site, NT-proBNP, and left ventricular ejection fraction (LVEF). Bold indicates statistical significance at *p *< 0.05

## Discussion

In this cross-sectional, population-based study, diabetes was significantly associated with elevated PWV/GLS, indicating a mismatch in VAC in the absence of established cardiovascular disease. Although the fully adjusted model did not show a statistically significant association between prediabetes and PWV/GLS, the significant relationship in the age- and sex-adjusted model, together with the linear association between HbA1c and VAC, suggests a gradual deterioration in VAC across the glycemic spectrum rather than a discrete shift occurring only at the diabetes threshold.

Several underlying mechanisms likely contribute to these findings in which diabetes, through activation of inflammation and matrix metalloproteinases, accelerates arteriosclerosis and thereby vascular stiffening [18–20]. As arterial stiffening progresses, PWV increases and shifts the time within the cardiac cycle at which pulse wave reflections return to the heart. Under normal conditions, the pulse wave generated by each heartbeat is reflected and returns to the heart during the diastolic phase, so it has minimal effect on left ventricular (LV) contraction [21, 22]. However, with elevated PWV, the pulse wave propagates faster through the arterial tree and arrives back at the heart during the systolic phase, when the LV is still contracting. This premature return elevates systolic pressure, prompting the LV to adapt by increasing its muscle mass through hypertrophy [23, 24]. Subsequently, elevated PWV has been shown to be highly associated with impaired LV strain, which is supposed to be attributed to an elevation of LV afterload imposed by stiffer arteries [25]. Moreover, LV longitudinal function has shown to be more sensitive to increased afterload compared to radial function, making GLS particularly susceptible to the impact of elevated PWV [26]. Thereby, the findings in the current study demonstrating higher PWV/GLS ratios in the presence of diabetes might be explained by the combined effects of arterial stiffening (reflected by increased PWV) and elevated left ventricular afterload (indicated by decreased GLS), ultimately leading to impaired VAC.

Extensive epidemiological and clinical studies indicate that, aside from other risk factors such as coronary artery disease (CAD) and hypertension, diabetes independently increases the risk of cardiac dysfunction [27]. This increased risk is believed to result from the harmful effects of chronic hyperglycemia on the myocardium, impairing cardiac function through both direct and indirect mechanisms, including disruptions in cell survival signaling, oxidative stress, and myocardial lipotoxicity [28]. Over time, these effects drive cardiac remodeling, presenting as both diastolic and systolic dysfunction, with systolic impairment—typically assessed by LV ejection fraction (LVEF)—emerging later during diabetes progression [29, 30]. Nonetheless, advanced imaging techniques such as tissue Doppler strain analysis and peak systolic velocity measurements have detected subtle systolic impairments in up to 24% of patients with diabetes, even after excluding those with CAD or left ventricular hypertrophy [31, 32].

While diabetes is a well-recognized risk factor for increased arterial stiffness and cardiac disease, the relationships may be bidirectional. Recent research indicates that heightened arterial stiffness could contribute to the development of type 2 diabetes, a risk that remains notably high even after accounting for established cardiovascular risk factors [33, 34].

This effect may be mediated by the impact of arterial stiffness on low-resistance organs, such as the pancreas, potentially exacerbating insulin resistance and further compromising glycemic control [35]. Consequently, impaired VAC could act as a central mediator in this feedback loop, and targeting VAC may offer the dual benefit of reducing cardiovascular risk and enhancing glucometabolic health in individuals with diabetes. This hypothesis is supported by evidence from an outcome trial comparing different treatment regimens for type 2 diabetes, including glucagon-like peptide-1 receptor agonists (GLP-1RA), sodium-glucose co-transporter 2 inhibitors (SGLT-2i), and insulin monotherapy. The study found that combination therapy with GLP-1RA and SGLT-2i led to a greater reduction in arterial stiffness and significant improvements in left ventricular function, as assessed by GLS. Furthermore, the combination therapy resulted in a substantial enhancement of VAC, measured by the PWV/GLS ratio [36]. These findings are consistent with the consensus recommendations that emphasize selecting medications which enhance VAC in patients with diabetes mellitus to improve clinical prognosis [1]. Our finding of higher eGFR in individuals with diabetes, alongside its gradual decline across increasing VAC quartiles, aligns with emerging evidence suggesting that certain glucose-lowering therapies may exert renoprotective effects, potentially preserving kidney function while modulating cardiovascular risk [7, 37].

Interestingly, although no significant association was observed between prediabetes and the PWV/GLS ratio in the fully adjusted model, a significant association was present in the age- and sex-adjusted analysis. This suggests that impairments in VAC may begin earlier in the glycemic spectrum and progress gradually rather than appearing abruptly at the threshold for overt diabetes. Several factors may explain the attenuation of the association after full adjustment. It is possible that individuals with prediabetes, despite showing early metabolic alterations, have not yet developed sufficient arterial stiffening to substantially affect VAC. Additionally, the use of antihypertensive medications—although not markedly higher among individuals with prediabetes compared to those with normoglycemia—may still have contributed to a blunted effect on both arterial stiffness and myocardial strain, potentially masking early VAC changes. The observed association between increasing HbA1c levels and elevated PWV/GLS, even in individuals without diagnosed diabetes, suggests that changes in VAC may begin early in the course of metabolic dysfunction. The lack of significant findings for prediabetes in our study might therefore be attributed to the limited sample size within this subgroup. This underscores the complexity of early metabolic alterations and the need for further research to elucidate the nuanced relationship between prediabetes, arterial stiffness, and VAC.

The VAC mismatch, as measured by PWV/GLS, has previously been shown to correlate strongly with heart failure (HF) severity, particularly in HF with preserved ejection fraction (HFpEF) [5]. In our study, conducted in a healthy population without overt HF or cardiovascular disease, we observed for the first time that diabetes is significantly associated with elevated PWV/GLS values. Additionally, our data indicate that higher PWV/GLS values are linked to increased measures of diastolic dysfunction, such as reduced septal e’ velocity and elevated E/e’ ratio as well as left ventricular hypertrophy. This raises the intriguing possibility that the increased risk of developing HFpEF in individuals with diabetes [38] may be mediated through impaired VAC, as reflected by PWV/GLS. In a previous study conducted in a young population-based cohort with a mean age of 28 years [39], a higher PWV/GLS ratio was associated with traditional cardiovascular risk factors such as hypertension, smoking, and obesity. Interestingly, no significant association was observed between prediabetes and PWV/GLS in that study, which may partly be explained by the relatively young age of the participants and the shorter duration of metabolic exposure. Our findings extend these observations by demonstrating a significant association between glycemic markers and VAC in a middle-aged population, supporting the notion that the impact of dysglycemia on VAC may become more apparent with advancing age and cumulative cardiometabolic burden. Interestingly, the inverse association observed between NT-proBNP and increasing PWV/GLS quartiles may reflect the vasodilatory effects of natriuretic peptides and aligns with recent population-based data [40], highlighting the need for further studies on the role of NT-proBNP in early VAC dysfunction.

### Strengths and limitations

Despite the compelling findings of our study, several limitations must be acknowledged. The cross-sectional design prevents the establishment of causality, highlighting the need for longitudinal studies to confirm the observed associations and clarify their temporal relationship. As this is a retrospective study utilizing pre-existing data, its findings are inherently constrained by the availability and quality of recorded variables, which may introduce selection bias or residual confounding. However, the SCAPIS cohort represents a uniquely well-characterized population sample with extensive phenotyping, which strengthens the validity of the analyses.

As SCAPIS is a population-based study predominantly including individuals without overt cardiovascular disease, and since we further excluded participants with known heart failure, prior myocardial infarction, or atrial fibrillation, the study population was relatively healthy. This may have limited the range of measured values and reduced the statistical power to detect associations at more extreme levels of cardiovascular impairment. Further, the study population was predominantly of Swedish-born descent, limiting the generalizability of our findings to other ethnic groups. Additionally, the inclusion of participants exclusively from the Malmö/Lund and Linköping sites, due to the unavailability of echocardiographic data from other SCAPIS centers, may restrict the applicability of our results to the entire cohort. The study experienced a substantial data loss, primarily due to challenges in obtaining high-quality strain imaging. Echocardiographic images were rigorously reviewed by an expert, who excluded cases where endocardial visualization was insufficient in multiple segments, ensuring the reliability of strain data but at the cost of reduced sample size. The inclusion of participants exclusively from the Malmö/Lund and Linköping sites, as echocardiographic data were not available from the other SCAPIS centers, which may limit the generalizability of our findings to the entire cohort. Additionally, the use of echocardiography and applanation tonometry to assess VAC may not capture all aspects of ventricular–arterial interaction, and future studies incorporating advanced imaging modalities are warranted. Also, while we adjusted for several confounding variables in our analysis, residual confounding cannot be entirely ruled out, and there may be unmeasured factors at the molecular and cellular levels. Data on specific pharmacological treatments, including antihypertensive drug classes and lipid-lowering therapies such as statins, were not available. This limits our ability to assess their potential confounding effects on the vascular and cardiac parameters studied. Advanced imaging techniques, such as cardiac magnetic resonance imaging and positron emission tomography, can provide detailed insights into the structural and functional changes occurring in the cardiovascular system in response to metabolic disturbances influencing the association between PWV/GLS and diabetes/prediabetes status.

In conclusion, we found an association between PWV/GLS and diabetes/prediabetes, that indicates VAC, measured using PWV/GLS may be a useful to assess cardiovascular risk stratification in these populations. Future research should focus on elucidating the underlying mechanisms linking VAC to metabolic disorders and exploring interventions aimed at improving VAC to reduce cardiovascular complications in high-risk individuals. Further, longitudinal studies are needed to determine whether VAC deterioration precedes cardiovascular events, serving as an early risk marker, or occurs as a consequence of worsening cardiometabolic health, further establishing its prognostic value and therapeutic potential.

## Electronic supplementary material

Below is the link to the electronic supplementary material.

Supplementary Material 1

## Data Availability

No datasets were generated or analysed during the current study.
