# Supplementary material for: Impact of diabetes and glycemic status on ventricular–arterial coupling in the general population
Source: Cardiovasc Diabetol. 2025 Apr 18;24:173. doi: 10.1186/s12933-025-02731-7 (PMC12008833; doi:10.1186/s12933-025-02731-7)
Supplement: Supplementary file 1 — Supplementary Material 1 [file 12933_2025_2731_MOESM1_ESM.docx]

**Supplementary table 1.** Population characteristics across quartiles of VAC (PVW/GLS) and post hoc pairwise comparisons between subgroups of PWV/GLS quartiles

|  | **All** | **Q1** | **Q2** | **Q3** | **Q4** | **P-value** | **P**  **Q1 vs Q2** | **P**  **Q1 vs Q3** | **P**  **Q1 vs Q4** | **P**  **Q2 vs Q3** | **P**  **Q2 vs Q4** | **P**  **Q3 vs Q4** |
| --- | --- | --- | --- | --- | --- | --- | --- | --- | --- | --- | --- | --- |
|  | **n=2884** | **n=729** | **n=726** | **n= 720** | **n= 709** |  |  |  |  |  |  |  |
|  |  | **0.28-0.47** | **0.47-0.53** | **0.53-0.62** | **0.62-1.25** |  |  |  |  |  |  |  |
| **Male sex, n (%)** | 1382 (48) | 170 (23) | 300 (41) | 403 (56) | 509 (72) | **<.001** | **<0.001** | **<0.001** | **<0.001** | **<0.001** | **<0.001** | **<0.001** |
| **Age, years (SD)** | 57 (4) | 56 (4) | 56 (4) | 57 (4) | 58 (4) | **<.001** | **0.027** | **<0.001** | **<0.001** | 0.017 | **<0.001** | 0.065 |
| **Systolic Blood Pressure, mmHg (SD)** | 81 (10) | 119 (14) | 126 (14) | 132 (14) | 141 (17) | **<.001** | **<0.001** | **<0.001** | **<0.001** | **<0.001** | **<0.001** | **<0.001** |
| **Diastolic Blood Pressure, mmHg (SD)** | 130 (17) | 75 (8) | 79 (9) | 82 (8) | 86 (10) | **<.001** | **<0.001** | **<0.001** | **<0.001** | **<0.001** | **<0.001** | **<0.001** |
| **Heart rate, beats/min (SD)** | 60 (9) | 58 (8) | 59 (7) | 60 (9) | 62 (9) | **<.001** | 0.712 | **<0.001** | **<0.001** | **0.004** | **<0.001** | **<0.001** |
| **Body Mass Index, kg/m^2 (SD)** | 26 (4) | 25 (3) | 25 (3) | 26 (3) | 27 (3) | **<.001** | 0.009 | **<0.001** | **<0.001** | 0.292 | **<0.001** | **<0.001** |
| **Height, cm (SD)** | 172 (9) | 168 (8) | 172 (9) | 174 (10) | 176 (9) | **<.001** | **<0.001** | **<0.001** | **<0.001** | **<0.001** | **<0.001** | **<0.001** |
| **Current Smoker, n (%)** | 291 (10) | 82 (11) | 70 (10) | 70 (10) | 69 (10) | .695 | 1 | 1 | 1 | 1 | 1 | 1 |
| **Hypertension, n (%)** | 1099 (38) | 137 (19) | 207 (29) | 286 (40) | 469 (66) | **<.001** | **<0.001** | **<0.001** | **<0.001** | **<0.001** | **<0.001** | **<0.001** |
| **Antihypertensive treatment, n (%)** | 112 (4) | 32 (4) | 25 (3) | 21 (3) | 34 (5) | **0.235** | 1 | 0.81 | 1 | 1 | 1 | 0.39 |
| **Classification of glycaemic status according to baseline examination results** |  |  |  |  |  |  |  |  |  |  |  |  |
| **Normoglycemia, n (%)** | 2388 (83) | 638 (88) | 622 (86) | 599 (83) | 529 (75) | **<.001** | 1 | 0.12 | **<0.001** | 1 | **<0.001** | **<0.001** |
| **Diabetes, n (%)** | 162 (6) | 26 (4) | 21 (3) | 39 (5) | 76 (11) | **<.001** | 1 | 0.534 | **<0.001** | 0.096 | **0.006** | **<0.001** |
| **Prediabetes, n (%)** | 334 (12) | 65 (9) | 83 (12) | 82 (12) | 104 (16) | **<.001** | 0.732 | 0.552 | **<0.001** | 1 | 0.084 | 0.138 |
| **Diabetes or prediabetes, n (%)** | 496 (17) | 91 (13) | 104 (14) | 121 (17) | 180 (25) | **<.001** | 1 | 0.12 | <0.001 | 1 | <0.001 | <0.001 |
| **Fasting plasma glucose, mmol/L (SD)** | 5. 7 (1.1) | 5.5 (0.7) | 5.6(0.9) | 5.7 (1.1) | 6.0 (1.5) | **<.001** | 0.072 | **<0.001** | **<0.001** | 0.230 | **<0.001** | **<0.001** |
| **HbA1c, mmol/mol (SD)** | 37 (8) | 35(4) | 35(5) | 36 (6) | 37 (8) | **<.001** | 0.958 | 0.313 | **<0.001** | 0.616 | **<0.001** | **<0.001** |
| **NT-proBNP, ng/L (SD)** | 60 (66) | 70 (58) | 60 (45) | 59 (51) | 59 (66) | **<.001** | 0.010 | **0.001** | **0.003** | 0.927 | 0.985 | 0.994 |
| **eGFR, mL/min/1.73 m^2 (SD)** | 76 (9) | 76 (9) | 75 (9) | 75 (8) | 74 (9) | **0.002** | 0.426 | 0.221 | **<0.001** | 0.978 | 0.082 | 0.199 |
| **LDL-C, mmol/L** | 3.4 (0.9) | 3.3 (0.9) | 3.4 (0.9) | 3.4 (0.9) | 3.5 (1.0) | **<.001** | 0.146 | 0.723 | 0.019 | 0.708 | 0.854 | 0.246 |
| **HDL-C, mmol/L** | 1.7 (0.5) | 1.9 (0.5) | 1.8 (0.5) | 1.7 (0.5) | 1.5 (0.5) | **<.001** | **<0.001** | **<0.001** | **<0.001** | **<0.001** | **<0.001** | **<0.001** |
| **Ejection Fraction, % (SD)** | 60 (5) | 61 (4) | 60 (4) | 59 (4) | 57 (4) | **<.001** | **<0.001** | **<0.001** | <0.001 | **<0.001** | **<0.001** | **<0.001** |
| **Mitral valve E-wave velocity, cm/s** | 66 (15) | 72 (15) | 67 (15) | 64 (14) | 61 (14) | **<.001** | **<0.001** | **<0.001** | **<0.001** | **<0.001** | **<0.001** | **<0.001** |
| **Septal e’, cm/s** | 6.9 (1.7) | 7.7 (1.7) | 7.2 (1.6) | 6.7 (1.6) | 6.1 (1.5) | **<.001** | 0.716 | 1 | **0.002** | 0.749 | 0.050 | **0.002** |
| **E/e’, unitless** | 9.9 (2.9) | 9.4 (2.5) | 9.6 (2.8) | 9.8 (3.0) | 10.2 (3.0) | **<.001** | 0.735 | 0.046 | **<0.001** | 0.373 | **<0.001** | 0.118 |
| **Left ventricular mass indexed (SD)** | 81 (20) | 72(17) | 73 (18) | 77 (19) | 81(20) | **<.001** | 0.351 | **<0.001** | **<0.001** | 0.008 | **<0.001** | **<0.001** |
| **Left ventricualr hypertrophy, n (%)** | 420 (15) | 64 (9) | 83 (11) | 111 (15) | 162 (23) | **<.001** | 0.558 | **<0.001** | **<0.001** | 0.156 | **<0.001** | **<0.001** |
| **Septal Hypertrophy, n (%)** | 428 (15) | 87 (12) | 92 (13) | 104 (15) | 145 (21) | **<.001** | 1 | 0.948 | **<0.001** | 1 | **<0.001** | 0.018 |
| **Septal e' < 7, n (%)** | 181 (6) | 36 (5) | 38 (5) | 45 (6) | 62 (9) | **.010** | 1 | 1 | 0.024 | 1 | 0.048 | 0.402 |
| **E/e' > 14, n (%)** | 200 (7) | 32 (5) | 45 (6) | 59 (9) | 64 (10) | **.003** | 1 | 0.024 | **<0.001** | 0.642 | 0.168 | 1 |

**eGFR**, estimated glomerular filtration rate; **HbA1c**, glycated hemoglobin; **NT-proBNP**, N-terminal pro-B-type natriuretic peptide; **e’**, Early diastolic mitral annular velocity; **E**, Early mitral inflow velocity; **LDL**, low density lipoprotein; **HDL**, high density lipoprotein
